# Supplementary material for: Construction of a depression risk prediction model for hepatitis B patients based on machine learning strategy
Source: PLoS One. 2026 Feb 4;21(2):e0341236. doi: 10.1371/journal.pone.0341236 (PMC12871976; doi:10.1371/journal.pone.0341236)
Supplement: Attachment — Submitted filename: Response to Reviewers.docx [file pone.0341236.s002.docx]

**Reviewer #1:**

1- Abstract:The use of abbreviation "NHANCE" without spelling in full. Please check as the abbreviation should be NHANES (National Health and Nutrition Examination Survey).

Thank you for the suggestion. I have systematically revised the entire text by replacing all instances of "NHANCE" with the standardized term "NHANES."

2- Introduction: The last paragraph includes a description of the methodology, which would be more appropriately placed in the Methods section.

We thank the reviewer for this valuable feedback. We have revised the manuscript accordingly by streamlining the methods-related content in the Discussion section as suggested.

3- Describe how Hep B was diagnosed to be entered in the database.

Thank you for the suggestion. Our inclusion specification details are as follows, and supplementary explanations have been provided in section 2, Method, 2.1 Data Acquisition. For details, please refer to the revised article: Serum samples from consenting NHANES participants were analyzed for three hepatitis B serological markers: anti-HBc (indicating past or present infection), HBsAg (suggesting current chronic or, rarely, acute infection), and anti-HBs (denoting vaccine-induced immunity or prior infection). Participants testing positive for anti-HBc were further categorized; those concurrently positive for HBsAg were classified as chronically infected. Individuals with anti-HBc-negative but anti-HBs-positive results were considered to have vaccine-induced immunity. The final analytical cohort comprised subjects with complete data for all three serological markers (anti-HBc, HBsAg, anti-HBs).

4- Any rationale why use random Forest for feature selection?

Thank you for this important question. In this study, we used Random Forest (RF) for feature selection, primarily based on its widespread application and recognized stability in medical prediction. As an ensemble of decision trees, RF has excellent capabilities for handling nonlinear relationships and multivariate interactions, making it particularly well-suited for complex and heterogeneous feature structures such as clinical data. Furthermore, during the modeling process, Random Forest naturally assesses the importance of each feature, providing a data-driven basis for variable selection and avoiding the bias associated with traditional methods that rely too heavily on manual judgment or univariate analysis. Furthermore, RF is robust to outliers and missing values and can handle high-dimensional, small-sample data, helping to improve model generalization and stability even with limited sample sizes. Therefore, we selected RF as a preliminary feature selection tool to improve prediction efficiency and provide a more informative variable set for subsequent multi-model construction.

5- I am not clear if dataset were split into training and test dataset. Describe clearly on this

We are grateful to the reviewer for pointing out this lack of clarity. In this study, we employed 5-fold cross-validation for appropriate dataset partitioning, training, and evaluation. Specifically, all HBV patient data included in the analysis were divided into five subsets. In each iteration, four of these subsets (approximately 80%) were used as training sets for model training and parameter optimization, while the remaining subset (approximately 20%) was used as a validation set to evaluate model performance. This process was repeated five times, ensuring that every sample participated in both training and validation, thereby comprehensively assessing the model's stability and generalization capabilities. 5-fold cross-validation not only improves efficiency with limited sample size but also effectively mitigates potential bias that might arise from a single partitioning of the model. During model evaluation, we aggregated performance metrics (such as AUC and F1-score) across all folds and averaged them as the final result. We will further clarify the description of the dataset partitioning and validation process in the revised manuscript. Thank you for your careful review.

6- Cross-validation strategy and hyperparameter tuning not explicitly described.

We thank the reviewer for their attention to methodological details. In this study, we systematically employed a five-fold cross-validation strategy throughout model construction and training, and performed hyperparameter tuning on the training set to improve model predictive performance and stability. Specifically, five-fold cross-validation was used to alternate training and validation between different subsamples to minimize performance fluctuations caused by sample splitting. For parameter settings, we performed preliminary grid search and manual tuning for each model. For example, in the Gradient Boosting model, we set 300 base learners (n_estimators=300) and controlled the learning rate (learning_rate=0.05) and tree depth (max_depth=4) to prevent overfitting. In the MLPClassifier, we used a three-layer neural network architecture with the ReLU activation function and the Adam optimizer, combined with L2 regularization (alpha=0.0001), and trained for a maximum of 500 iterations to ensure convergence and robustness. For Logistic Regression, we introduced an L2 regularization term and set the maximum number of iterations to 1000. Throughout the training process, we strictly used the training set for model fitting and the validation set for performance evaluation, and no data leakage occurred. We will further refine the relevant descriptions in the methods section of the paper to improve the transparency and reproducibility of the research.

7- Providing a summary of the final sample’s sociodemographic characteristics would greatly enhance understanding of the dataset. It is also unclear whether the final sample includes only adults or also includes children—this should be clarified.

We appreciate the reviewer's valuable feedback. In response, we have conducted comprehensive statistical analyses of the data and incorporated a detailed sociodemographic characteristics section into the manuscript. The added content is summarized below and presented in Section 3 (Results), Subsection 3.1 (Sociodemographic Characteristics) of the revised manuscript. Please refer to the updated Section 3.1 for complete details: This study included 189 patients diagnosed with hepatitis B. The demographic characteristics are summarized as follows: the mean age was 49.03 ± 16.12 years; 107 patients (56.61%) were male and 82 (43.39%) were female. An analysis of marital status, based on an effective sample of 183 participants (missing data: 6), revealed that 105 (57.38%) were married, 22 (12.02%) were widowed, 20 (10.93%) were divorced, 5 (2.73%) were separated, and 30 (16.39%) had never been married. Regarding educational attainment, based on an effective sample of 182 participants (missing data: 7), 86 patients (47.25%) had less than postsecondary education, while 96 (52.75%) had attained postsecondary education or higher.

8- Consider limiting discussion statements in the Results section, as it currently includes substantial discussion that would be more appropriately addressed in the Discussion section

We sincerely appreciate the valuable feedback provided by the reviewer. In accordance with your suggestion, we have rigorously focused the presentation of findings in the Results section, while significantly expanding the discussion of clinical implications in the Discussion section to provide a more robust interpretation of our outcomes.

9- It is advisable to present the results separately for the training and test datasets to facilitate comparison and assess model performance. This is particularly important given that overfitting is a common concern in machine learning algorithms.

We strongly agree with the reviewer's suggestion that model performance should be presented separately on the training set and the test set. Taking into account the relatively limited sample size of this study, we adopted a five-fold cross-validation strategy, so the training set and validation set are not statically divided at one time, but are dynamically rotated. In each fold, the training model is only trained on 80% of the subsamples, while 20% of the subsamples are independently used for validation. The performance indicator after five rounds of iteration is the average test effect. Since the training and test sets are different in each round, reporting the results of each fold separately may lead to confusion in interpretation. Therefore, we regard the overall indicator of the five-fold validation as the robustness of the model on the "test set". The performance indicators during the training process are not used as the basis for evaluation, mainly to avoid the model from masking the generalization problem due to good performance on the training set.

10- While the MLPClassifier achieved high accuracy and strong performance metrics, the poor calibration curve may indicate overfitting or that the model's predicted probabilities do not align well with actual outcomes.??

Thank you for the reviewer's attention to the calibration performance of MLPClassifier. We did observe that although the model performed well in classification performance indicators such as AUC and Recall, its probability calibration curve deviated from the ideal diagonal line, suggesting that its predicted probabilities did not perfectly match the true labels. One possible reason for this phenomenon is that the neural network model itself tends to output probability distributions "overconfidently", especially when the sample size is limited and the class imbalance is unbalanced (corrected using SMOTE). This output probability deviation is a common phenomenon in deep learning models, but it does not necessarily mean that the model as a whole is severely overfitted. Our main goal is to identify high-risk groups with high sensitivity, so we mainly choose models with higher Recall. In practical applications, the calibration problem can be corrected through post-processing methods such as Platt scaling or Isotonic regression, and we will consider introducing such technologies in subsequent studies.

11- Overall Calibration plots suggest poor calibration despite high AUC??

We understand and appreciate the reviewer's cautious assessment of model calibration performance. We did note that while the models demonstrated good discriminative ability in metrics such as AUC and F1-score, the calibration curves of some models (particularly the MLPClassifier) deviated somewhat from the ideal curve. This suggests that while the model is able to distinguish positive and negative samples well, the probability values it outputs may be somewhat exaggerated in interpretation, manifesting as "unconservative probabilities." We believe this is a common technical issue in deep learning models, particularly under limited sample conditions, where models struggle to calibrate equally across all probability intervals. It is important to emphasize that we employed regularization (such as L2 penalty and dropout) and cross-validation during model construction to minimize overfitting, demonstrating the potential for practical application in risk stratification. We plan to further introduce calibration mechanisms to improve model interpretability, and we thank the reviewer for their expert attention to this issue.

12- Include discussion on model overfitting.

We thank the reviewers for raising concerns about the risk of model overfitting. We fully agree that when using machine learning methods, especially deep learning models, the generalization ability of the model must be fully discussed and carefully evaluated. Therefore, in the "Limitations" section of the Discussion section, we have added a systematic analysis of the overfitting issue, explicitly emphasizing that the high performance achieved by the current model (particularly the MLPClassifier) under small sample conditions (e.g., Recall = 0.980, AUC = 0.935) should be interpreted with caution. In our discussion, we pointed out that despite using SMOTE to mitigate class imbalance and incorporating conventional strategies such as L2 regularization and depth control to constrain model complexity, the model still faces the risk of "learning incidental patterns in the data" in the context of limited NHANES data and sample size, thereby overestimating its applicability to real-world clinical scenarios. We further point out that deviations from the calibration curve can also serve as evidence of potential overfitting. Furthermore, we emphasize that future studies should prioritize external independent validation, particularly in larger, multicenter, prospective cohorts, to truly assess the model's generalizability and stability in clinical settings.

13- Limitation of small sample size/ likely cross-sectional dataset

We appreciate the reviewer's insightful comments regarding the limitations associated with the modest sample size and cross-sectional nature of our dataset. These methodological constraints have been explicitly addressed in the revised Discussion section (pages 12). We acknowledge these limitations and propose that future studies with larger multi-center prospective cohorts and external validation would strengthen the generalizability of our findings.

14- Potentially overstated claims about model performance and clinical utility...may need in-depth explanation/discussion

We sincerely appreciate the reviewer's valuable feedback. In response, we have revised the descriptions of model performance and clinical utility throughout the manuscript, carefully modifying any statements that could be perceived as overstated claims. We have also expanded the discussion addressing these critical aspects, with comprehensive amendments visible in the revised Discussion section.

15- The strong performance metrics observed, despite the limited sample size, may be overly optimistic and warrant caution when interpreting the model’s reliability.

We are grateful for this insightful comment. In light of your feedback, we have rigorously re-examined the reported findings and observed metrics through a more critical scientific lens. The robustness of our model has been interpreted with appropriate caution, and the Discussion section now provides substantive analysis of both methodological strengths and limitations.

16- If feasible, obtaining a larger sample and rerunning the analysis would enhance the robustness and generalizability of the findings.

We sincerely appreciate the insightful feedback regarding methodological limitations. We fully acknowledge the constraints inherent in our study's limited sample size and cross-sectional dataset. To address these limitations, we are actively planning a large-scale, multicenter, prospective external validation study in future research.

**Reviewer #2:**

17- How many participants with hepatitis B viruses were recruited in your study?

We sincerely appreciate the reviewer's valuable comments. In response, we have conducted a comprehensive reorganization and analysis of the demographic information for our study cohort. The results demonstrate that the study ultimately enrolled 189 hepatitis B patients. Additional details regarding population characteristics are presented in Section 3 (Results), specifically subsection 3.1 (Sociodemographic Characteristics) of the revised manuscript, which has been substantially expanded to address this point.

18- Data preprocessing and feature screening
    For numerical variables, the mean was used for filling. but, the main drawback of using the mean is its sensitivity to outliers or extreme values in the dataset. These extreme values can disproportionately affect the mean, making it a potentially misleading representation of the typical value, especially with small sample sizes. So do you generalize with this limitation?

Thank you for raising important questions about missing value imputation methods. We fully understand your concern that mean imputation may be affected by extreme values, especially in small sample sizes. During data preprocessing, we used the mean to impute numeric variables for two main reasons: First, the dataset we used (NHANES) is a structured and standardized data source with rigorous quality control during data collection and processing, and the number of extreme outliers is relatively limited; second, in the context of small sample sizes, introducing more complex or parameterized imputation methods (such as KNN and MICE) may introduce additional variance or computational bias, especially in high-dimensional feature spaces, which can affect model stability.

At the same time, we removed significant outliers during data cleaning to mitigate the risk of mean imputation being affected by extreme values. We are also fully aware that mean imputation is not suitable for all situations and does have limitations, such as sensitivity to extreme values and inability to preserve data distribution characteristics. Therefore, the current imputation strategy is a compromise between balancing sample size and model complexity. In the future, based on the expansion of sample size or richer data dimensions, more robust multiple imputation or model-driven imputation methods can be introduced to further improve data quality and the generalization ability of the prediction model.

19- Your discussion at all is not supported by the existing evidences. i.e it lacks references. Please rewrite it.

We express our sincere gratitude for your invaluable feedback. Substantial revisions have been made to the Discussion section, incorporating multiple new references. Our revised discussion significantly enhances scientific rigor by providing in-depth pathophysiological explanations that mechanistically link key predictors to depression pathogenesis in hepatitis B patients, with robust support from existing literature. We have implemented rigorous model performance evaluation, explicitly acknowledging potential overfitting risks stemming from the limited sample size (n=189) and SMOTE oversampling, despite strong internal metrics (AUC = 0.935, recall = 0.980).

Furthermore, limitations are now thoroughly delineated with proposed resolutions: 1) external validation through multicenter prospective cohorts, 2) integration of multimodal neurobiological/omics data, and 3) implementation of explainable AI (XAI) to enhance clinical transparency. Conclusions have been appropriately tempered regarding claims of clinical utility pending further validation, thereby strengthening biological plausibility, methodological honesty, and the translational pathway.
