# Supplementary material for: Construction of a depression risk prediction model for hepatitis B patients based on machine learning strategy
Source: PLoS One. 2026 Feb 4;21(2):e0341236. doi: 10.1371/journal.pone.0341236 (PMC12871976; doi:10.1371/journal.pone.0341236)
Supplement: Attachment — Submitted filename: Response_to_Reviewers_auresp_2.docx [file pone.0341236.s003.docx]

Dear Editor

We sincerely thank the Editor and the Reviewers for their time and valuable comments on our manuscript. In this rebuttal letter, we provide a point-by-point response to each comment. Specifically, for constructive suggestions, we have implemented revisions directly in the manuscript. For concerns regarding methodological conception and inherent limitations, we provide detailed explanations below, supported by evidence from the peer-reviewed literature, to justify the rationale and validity of our approach.

1、clinical application prospects of the model

We sincerely appreciate Reviewer 1's valuable suggestions. We fully agree that incorporating the clinical application prospects of the model will enhance the completeness of the manuscript. Accordingly, we have added a new paragraph in the Discussion section (Page 14, Line 67) to specifically explore the potential application scenarios and value of our model in future clinical practice, as well as the challenges and subsequent research directions required for its translation into a practical tool, as detailed in the text.

2、We thank the reviewer for raising these important points. We would like to respectfully address these concerns by clarifying the methodological choices made in our study.

Firstly, regarding **methodological rigor, o**ur analytical pipeline was constructed using established and robust practices. We employed a diverse set of five machine learning algorithms (Gradient Boosting, Logistic Regression, AdaBoost, MLPClassifier, LDA), each selected for its complementary strengths. To ensure generalizability and mitigate overfitting, we implemented a rigorous 5-fold cross-validation framework and performed careful hyperparameter tuning for each model. Furthermore, to enhance the interpretability of our models, we conducted a feature importance analysis, which provides insights into the key predictors and aligns with the objective of explaining model decisions. The consistent high performance across multiple models, particularly the non-linear classifiers, underscores the robustness of our approach.

Secondly, regarding data sufficiency, the model was developed using a well-characterized cohort of 189 patients with Hepatitis B, derived from the NHANES database—a nationally representative and widely recognized resource. While larger sample sizes are generally advantageous, the cohort size in this study is substantial for a machine learning investigation focused on a specific patient subpopulation. Furthermore, it is directly comparable to sample sizes employed in previously published studies; for instance, studies by Cui et al.^[^^[1]]^ and Cao et al. ^[^^[2]]^utilized cohorts of 239 stroke patients and 309 patients with infertility, respectively. The quality and breadth of the NHANES data, which encompasses comprehensive demographic, laboratory, and clinical examination variables, provide a robust foundation for model development.

Finally, on the point of **model interpretability**, we proactively addressed the "black box" concern by implementing a **two-stage interpretability strategy**. First, we used the **Random Forest algorithm** to perform robust feature selection, identifying the top 20 predictive features, which directly highlights the most influential variables for clinical interpretation (Figure 2). Second, the strong performance of inherently more interpretable models like **Gradient Boosting** (AUC=0.919), which provides built-in feature importance metrics, further validates the relevance of the selected features. The identified predictors including liver function markers, electrolyte levels, inflammatory indices, and socioeconomic factors are all **clinically plausible and supported by existing literature** on depression and chronic liver disease, thereby enhancing the biological and psychological interpretability of our findings. Also, this approach to interpretability is well-established and widely accepted in the literature for clinical risk prediction models^[^^[3][^^[4]][^^[5]]^.

Therefore, we believe that the methodology adopted in our study is sound, the data is sufficient for its purpose, and the model offers a reasonable and interpretable framework for risk assessment.

3、We sincerely appreciate the reviewer's insightful comment regarding the use of cross-sectional data for predictive modeling. We acknowledge that a prospective longitudinal design is the ideal and most rigorous standard for establishing definitive causal relationships and predicting future incident events.

However, the development and validation of so-called "predictive models" using cross-sectional data represents a well-established and valuable methodological approach in exploratory research, particularly when leveraging large-scale public databases such as the one utilized in our study. The primary objective in this context is not to infer causality or to forecast future events with certainty, but to identify a robust set of features strongly associated with the prevalent outcome, thereby estimating an individual's current risk profile or susceptibility. This approach serves as a critical first step for risk stratification and hypothesis generation, which subsequently warrant validation in longitudinal cohorts. We indeed plan to initiate such a cohort study as a next step.

Crucially, this paradigm is widely accepted and has been extensively employed in high-quality literature. For instance, the research conducted by Yan et al., which developed a model for predicting chronic kidney disease risk using NHANES data^[^^[6]]^; the research conducted by Huang and Liu, which created a stroke risk assessment tool based on the NHANES database^[^^[7]]^; the research conducted by Zheng et al., which built a machine learning model to predict osteoarthritis risk from volatile organic compound exposure using NHANES data^[^^[8]]^; the research conducted by Zhang et al., which developed an XGBoost-based prediction model for metabolic dysfunction-associated steatotic liver disease using NHANES data^[^^[9]]^; the research conducted by Gong et al., which created an explainable AI model for rapid diagnosis of COVID-19 using ensemble learning algorithms^[^^[10]]^; and the research conducted by Ahiduzzaman et al., which established an interpretable machine learning framework for cardiovascular risk prediction using NHANES dietary and health data, collectively demonstrate the feasibility and scientific merit of this approach^[^^[11]]^.

To precisely address the reviewer's valid point and to ensure the most accurate terminology, we have opted to refine our manuscript's wording. We have also further elaborated on this specific limitation in the discussion section, explicitly stating that future longitudinal studies are required to confirm the temporal relationship and true predictive capacity.

We acknowledge that models developed exclusively from cross-sectional data, such as NHANES, are more accurately characterized as "discriminative models" or "diagnostic models" rather than prospective "prognostic prediction models." In our study (and in similar cited research on hepatitis B and depression), the objective of the model is to utilize clinical indicators collected at a single time point to identify or distinguish those hepatitis B patients who currently have depression but may not yet be clinically diagnosed. We fully agree that establishing a model to predict the future risk of incident depression in hepatitis B patients necessitates a prospective cohort design. We have explicitly addressed this limitation in the discussion section (Section 4), stating that cross-sectional data cannot support causal inference and recommending validation through external prospective cohorts in future studies. Consequently, the term "prediction," as used in our manuscript, primarily operates within the "input-output" context of machine learning, referring to the process of estimating an unknown status based on known information. We appreciate this review, which has heightened our awareness of the need for more precise definitions of "prediction" in future research and scientific communication to prevent ambiguity.

In summary, we have thoroughly addressed the reviewers' comments to the best of our ability, and including a new discussion on the clinical applications of our model. We believe our study presents a valuable and methodologically sound framework for assessing depression risk in patients with Hepatitis B by leveraging multi-dimensional data. While we have contextualized the inherent limitations of cross-sectional design with support from the published literature, the core clinical insights and methodological rigor of our work remain robust and informative. We are enthusiastic about the potential of our research to contribute to improved mental health management for patients with Hepatitis B. Thank you again for your consideration and for providing the opportunity to revise. We sincerely hope that you find our responses satisfactory and that the manuscript is now deemed suitable for publication in **PLOS ONE**.

**References**

1. ^[]^ Cui Y, Gao J, Zhang J, Li J, Wang Z, Xu S, Zhao J. Machine learning prediction of thrombolysis efficacy using hs-CRP and inflammatory markers in stroke. Medicine (Baltimore). 2025 Oct 24;104(43):e45405. doi: 10.1097/MD.0000000000045405IF: 1.4 Q2 . PMID: 41137332; PMCID: PMC12558252.
2. ^[]^ Cao S, Cai M, Zhang H, Huang H, Xu L, Lin N. Association between high-density lipoprotein cholesterol-related inflammatory markers and female infertility: a cross-sectional analysis from the National Health and Nutrition Examination Survey (NHANES 2013-2018). BMC Womens Health. 2025 Oct 21;25(1):503. doi: 10.1186/s12905-025-03917-7. PMID: 41120997; PMCID: PMC12538855.
3. ^[]^ Zhou Z, Ding Q, Tang X, Han L, Wang Y, Qian J, Li K, Zhou Q. Insulin Resistance Indices Predict Mortality in Cardiovascular Disease: A Large-Scale NHANES Study With Machine Learning Validation. Food Sci Nutr. 2025 Nov 19;13(11):e71080. doi: 10.1002/fsn3.71080. PMID: 41268094; PMCID: PMC12628084.
4. ^[]^ Feng T, Ou Q, Shan G, Hu Y, He H. A predictive model for metabolic syndrome in a community-based population with sleep apnea: a secondary prevention screening tool using simple and accessible indicators. Front Nutr. 2025 Nov 5;12:1667055. doi: 10.3389/fnut.2025.1667055. PMID: 41267995; PMCID: PMC12626801.
5. ^[]^ Yu ZC, Shi ZJ, Fang ZK, Liu SY, Yu Y, Wang KD, Huang DS, Shen GL, Zhang CW, Liang L. Development and validation of machine learning-based model for predicting early recurrence for patients with HBV-associated hepatocellular carcinoma after curative hepatectomy. Am J Surg. 2025 Nov 8;251:116716. doi: 10.1016/j.amjsurg.2025.116716. Epub ahead of print. PMID: 41265205.
6. ^[]^ Yan B, Yuan R, Yin L, Huang S. Age-adjusted visceral adiposity index as a predictor of chronic kidney disease: insights from NHANES 2007-2018. Ren Fail. 2025 Dec;47(1):2578412. doi: 10.1080/0886022X.2025.2578412IF: 3.0 Q1 . Epub 2025 Nov 4. PMID: 41188187; PMCID: PMC12587790.
7. ^[]^ Huang J, Liu W. Development and validation of a machine learning model to predict stroke risk based on the NHANES database. Medicine (Baltimore). 2025 Nov 7;104(45):e45800. doi: 10.1097/MD.0000000000045800. PMID: 41204470; PMCID: PMC12599744.
8. ^[]^ Zheng S, Zhu J, Cao X, Chen Z, Zhang C, Xia T, Shen J. Machine learning prediction of osteoarthritis risk from volatile organic compound exposure using SHAP interpretation in US adults. Sci Rep. 2025 Nov 11;15(1):39477. doi: 10.1038/s41598-025-23050-7. PMID: 41219301; PMCID: PMC12606308.
9. ^[]^ Zhang Y, Liu X, Zhang X, Fei Y, Li X. Machine learning-based prediction of metabolic dysfunction-associated steatotic liver disease using National Health and Nutrition Examination Survey (NHANES) data. PLoS One. 2025 Nov 12;20(11):e0335656. doi: 10.1371/journal.pone.0335656. PMID: 41223197; PMCID: PMC12611120.
10. ^[]^ Ahiduzzaman M, Hasan MN. Interpretable machine learning for cardiovascular risk prediction: Insights from NHANES dietary and health data. PLoS One. 2025 Nov 6;20(11):e0335915. doi: 10.1371/journal.pone.0335915. PMID: 41196937; PMCID: PMC12591444.
11. ^[]^ Zhang Y, Liu X, Zhang X, Fei Y, Li X. Machine learning-based prediction of metabolic dysfunction-associated steatotic liver disease using National Health and Nutrition Examination Survey (NHANES) data. PLoS One. 2025 Nov 12;20(11):e0335656. doi: 10.1371/journal.pone.0335656. PMID: 41223197; PMCID: PMC12611120.
